# Downregulation of miR-610 promotes proliferation and tumorigenicity and activates Wnt/β-catenin signaling in human hepatocellular carcinoma

**DOI:** 10.1186/1476-4598-13-261

**Published:** 2014-12-10

**Authors:** Xian-Cheng Zeng, Fo-Qiu Liu, Rong Yan, Hui-Min Yi, Tong Zhang, Guo-Ying Wang, Yang Li, Nan Jiang

**Affiliations:** Department of General Surgery, Zengcheng People’s Hospital, (BoJi-Affiliated Hospital of Sun Yat-Sen University), Zengcheng, Guangdong China; Department of clinical laboratory, Zengcheng People’s Hospital (BoJi-Affiliated Hospital of Sun Yat-Sen University), Zengcheng, Guangdong China; Department of Hepatic Surgery, The Third Affiliated Hospital of Sun Yat-sen University, 600 Tian He Road, Tian He District, Guangzhou, Guangdong 310630 China; Department of Gastroenterology, Zengcheng People’s Hospital (BoJi-Affiliated Hospital of Sun Yat-Sen University), Zengcheng, Guangdong China

**Keywords:** HCC, miR-610, Proliferation, Wnt/β-catenin, LRP6, TBL1X

## Abstract

**Background:**

Wnt/β-catenin signaling pathway plays important roles in human cancer progression. Better understanding the mechanism underlying regulation of Wnt/β-catenin signaling pathway might provide novel therapeutic targets for cancer treatment.

**Methods:**

miR-610 expression levels in hepatocellular carcinoma (HCC) cell lines, HCC tissues and 76 archived HCC specimens were determined using real-time PCR. Cell viability was measured by 3-[4, 5-dimethylthiazol-2-yl]-2, 5-diphenyltetrazolium bromide (MTT) assay. The level of DNA synthesis was determined by BrdU incorporation assay. Flow cytometry analysis was used to analyze cell cycle progression. The cells proliferation and tumorigenesis were determined by colony formation and anchorage-independent growth assays *in vitro*, and by xenograft tumors *in vivo*. Luciferase assay and micro-ribonucleoprotein complex immunoprecipitation assay were used to confirm the association of the targeted mRNAs with miR-610.

**Results:**

miR-610 was downregulated in human HCC cells and tissues, and correlated with HCC progression and patient survival. Inhibition of miR-610 promoted, but overexpression of miR-610 reduced, HCC cell proliferation and tumorigenicity both *in vitro* and *in vivo*. Furthermore, we found that inhibiting miR-610 activated, but overexpressing miR-610 decreased, the Wnt/β-catenin activity through directly suppressing lipoprotein receptor-related protein 6 (LRP6) and transducin β–like protein 1 (TBL1X). The *in vitro* analysis was consistent with the inverse correlation detected between *miR-610* levels with expression of LRP6 and TBL1X in a cohort of human HCC samples.

**Conclusions:**

Our results indicate that miR-610 downregulation plays essential roles in HCC progression and reduced miR-610 is correlated with Wnt/β-catenin signaling pathway.

**Electronic supplementary material:**

The online version of this article (doi:10.1186/1476-4598-13-261) contains supplementary material, which is available to authorized users.

## Background

Hepatocellular carcinoma (HCC) is the fifth most prevalent cancer and the third leading cause of cancer mortality worldwide [1–3]. Although the clinical course and survival rates in HCC depend on the disease stage at diagnosis, most patients are initially diagnosed at the advanced stages, there is no effective therapeutic treatment, resulting in short survival time and poor prognosis [4, 5]. Poor understanding of the mechanisms underlying HCC pathogenesis renders early-stage diagnosis and treatment difficult [6]. Therefore, further studies are needed to investigate the progression of HCC initiation and pathogenesis, which would contribute to the exploration of effective schemes for HCC diagnosis and therapy.

Wnt/β-catenin signaling pathway is highly conserved in evolutionary processes and reported to be overactivated in the progresses of multiple tumors, including HCC [7–14]. The varying distribution of β-catenin in tumor cells leads the abnormal activation of Wnt/β-catenin signaling pathway correlated with cancers prognosis [11, 14–16]. Satoshi *et al.* reported that β-catenin plays essential roles in promoting HCC progression by stimulating HCC cell proliferation and suppressing cell adhesion, and is associated with a poor prognosis of HCC patients [14]. Furthermore, Wnt/β-catenin signaling pathway modulates multiple genes correlated with tumor progression, such as Cyclin D, Ki67, and E-cadherein [14]. Therefore, it is of great interest to investigate the regulatory mechanism of Wnt/β-catenin signaling pathway in HCC and it might be potential target for HCC diagnosis and therapy.

As a member of the expanding low-density lipoprotein (LDL) receptor family, lipoprotein receptor-related protein 6 (LRP6), is found to be expressed in different types of human tissues and LRP6 is one of Wnt-coreceptors, which could activate the transcription of Wnt/β-catenin target genes by promoting β-catenin translocation into the nucleus [17–19]. Meanwhile, LRP6 is also found to be correlated with cancer initiation and progression and significantly overexpressed in various types of human cancers, such as liver cancer, colon cancer, and kidney tumor [20, 21]. In HCC, LRP6 is also reported to be upregulated and overexpression of LRP6 enhanced HCC cells proliferation, migration and invasion [22]. It has been reported that transducin β–like protein 1 (TBL1X) and its highly related family member TBLR1 could bind to the E3 ubiquitin ligase components SIAH-1 and SKP1 to inhibit β-catenin degradation leading to the activation of Wnt/β-catenin signaling and TBL1-TBLR1and β-catenin recruit each other to Wnt target-gene promoter for transcription activation and oncogenesis [23]. Depletion of TBL1–TBLR1 inhibited Wnt-β-catenin-induced gene expression and oncogenic growth [23, 24]. Therefore, it would be interesting to investigate the regulatory mechanism of LRP6 or TBL1X in HCC.

MicroRNAs (miRNAs), a class of small noncoding RNAs, are important elements in numerous biological activities and modulation of multiple cellular processes through negative regulation of gene expression by targeting the 3’ untranslated region (3’ UTR) of specific mRNAs in a sequence-specific manner [25–27]. Aberrant miRNAs expressions have been implicated in the initiation and progression of various tumors and plays vital roles in tumor development [28–37].

In the present study, we reported that miR-610 was downregulated in HCC cell lines and tissues. Inhibition of miR-610 promoted, while upregulation of miR-610 suppressed, HCC cell proliferation and tumorigenicity both *in vitro* and *in vivo*. Furthermore, we demonstrated that miR-610 inhibited Wnt/β-catenin signaling activity through directly downregulation of LRP6 and TBL1X. Therefore, our results suggest that miR-610 might play important functions in HCC progression and represent a potential target for HCC diagnosis and therapy.

## Results

### MiR-610 is downregulated in HCC and correlated with progression and survival

**Figure 1:**
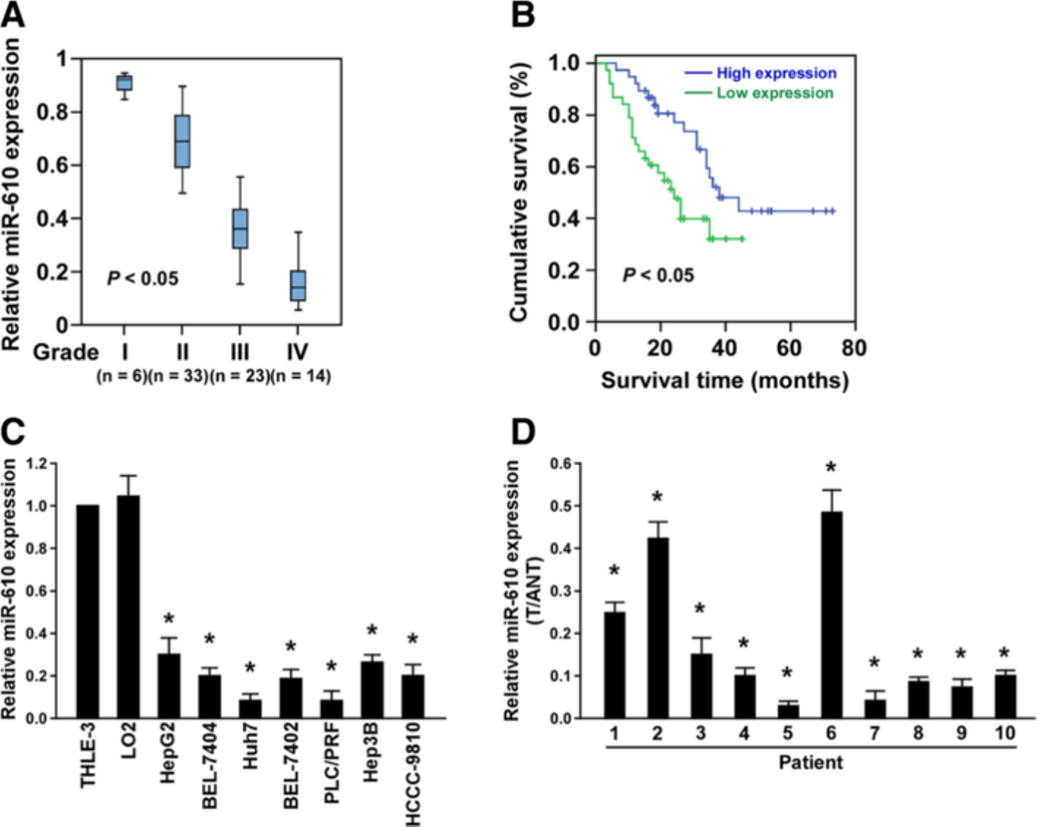
**MiR-610 is downregulated in HCC and correlates with HCC progression and survival. A**, Correlation between miR-610 expression in different grades of HCC assessed by real-time PCR. Box boundaries represent lower and upper quartiles, respectively; lines within boxes and whiskers denote median and extremum, respectively. **B**, Correlation between miR-610 levels and overall survival by Kaplan–Meier analysis of patients with low (<median) or high (>median) miR-610 expression. **C**, MiR-610 expression levels in HCC cell lines compared with normal human hepatic cells. **D**, Comparison of miR-610 levels in 10 paired HCC tissues (T) and their adjacent noncancerous tissues (ANT). Average miR-610 expression was normalized using U6 expression. Bars represent the means ± SD of three independent experiments. *P <0.05.

By analyzing the published, microarray-based high-throughput assessment (NCBI/GEO/GSE31384, n =166; *P* <0.05), miR-610 was found to be significantly downregulated in HCC tissue compared with the matched noncancerous tissue (Additional file 1: Figure S1). To assess whether miR-610 downregulation is linked to HCC progression, we analyzed miR-610 levels in 76 archived clinical HCC specimens. As shown in Figure 1A, miR-610 levels remained high in grade I tumors but were markedly lower in grade II, III and IV tumors. Statistical analysis revealed that miR-610 levels were inversely correlated with HCC progression (*P* <0.05; Additional file 2: Table S1 and S2). Importantly, reduced miR-610 expression was closely associated with shorter overall survival time (*P* <0.05) (Figure 1B, Additional file 2: Table S2). Consistently, real-time PCR analysis revealed significant downregulation of miR-610 expression in all seven HCC cell lines compared with the two normal hepatic cell lines (Figure 1C) and in the 10 HCC tissues compared with the paired adjacent noncancerous tissues (Figure 1D). Collectively, these results suggest that miR-610 is downregulated in HCC and reduced miR-610 might represent as a predictive biomarker for HCC diagnosis and prognosis.

### Inhibition of miR-610 enhances HCC cell proliferation and cell cycle progression

**Figure 2:**
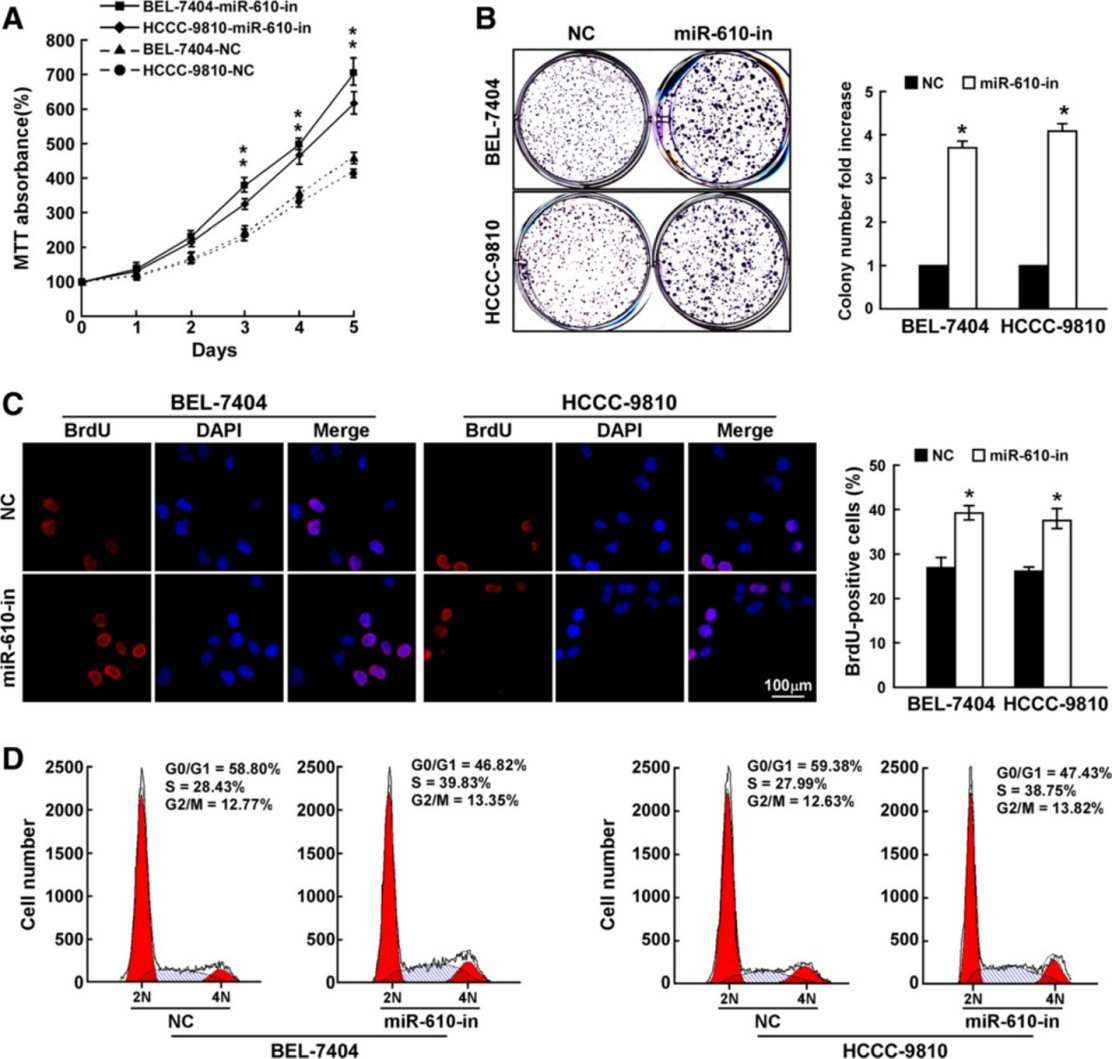
**MiR-610 inhibition enhances HCC cell proliferation and cell cycle progression. A**, MTT analysis of cell growth rates of cell lines transfected with miR-610 inhibitor (miR-610-in) or negative control (NC) after seeding. **B**, Representative micrographs (left) and quantification (right) of HCC cell colonies determined by colony formation assay. **C**, Representative micrographs (left) and quantification (right) of BrdU incorporation in HCC cells. **D**, Flow cytometry analysis of cell cycle progression in HCC cells. Bars represent the means ± SD of three independent experiments. *P <0.05.

To further explore the biological role of reduced miR-610 in HCC progression, MTT and colony formation assays were performed and results of both assays revealed that inhibition of miR-610 dramatically promoted proliferation of HCC cells compared with that of control cells (Figure 2A and B, Additional file 3: Figure S2). Furthermore, BrdU incorporation assay showed that miR-610–inhibited cells displayed higher DNA synthesis levels than that in control cells (Figure 2C). Consistent with these findings, cell cycle analysis using flow cytometry assay showed a decreased percentage of G1/G0-phase cells and an increased percentage of S-phase cells (Figure 2D). These results indicate that miR-610 inhibition leads to the promotion of HCC cell proliferation, which suggest that miR-610 might function as a tumor suppressor.

### Upregulation of miR-610 suppresses HCC cell proliferation and cell cycle progression

**Figure 3:**
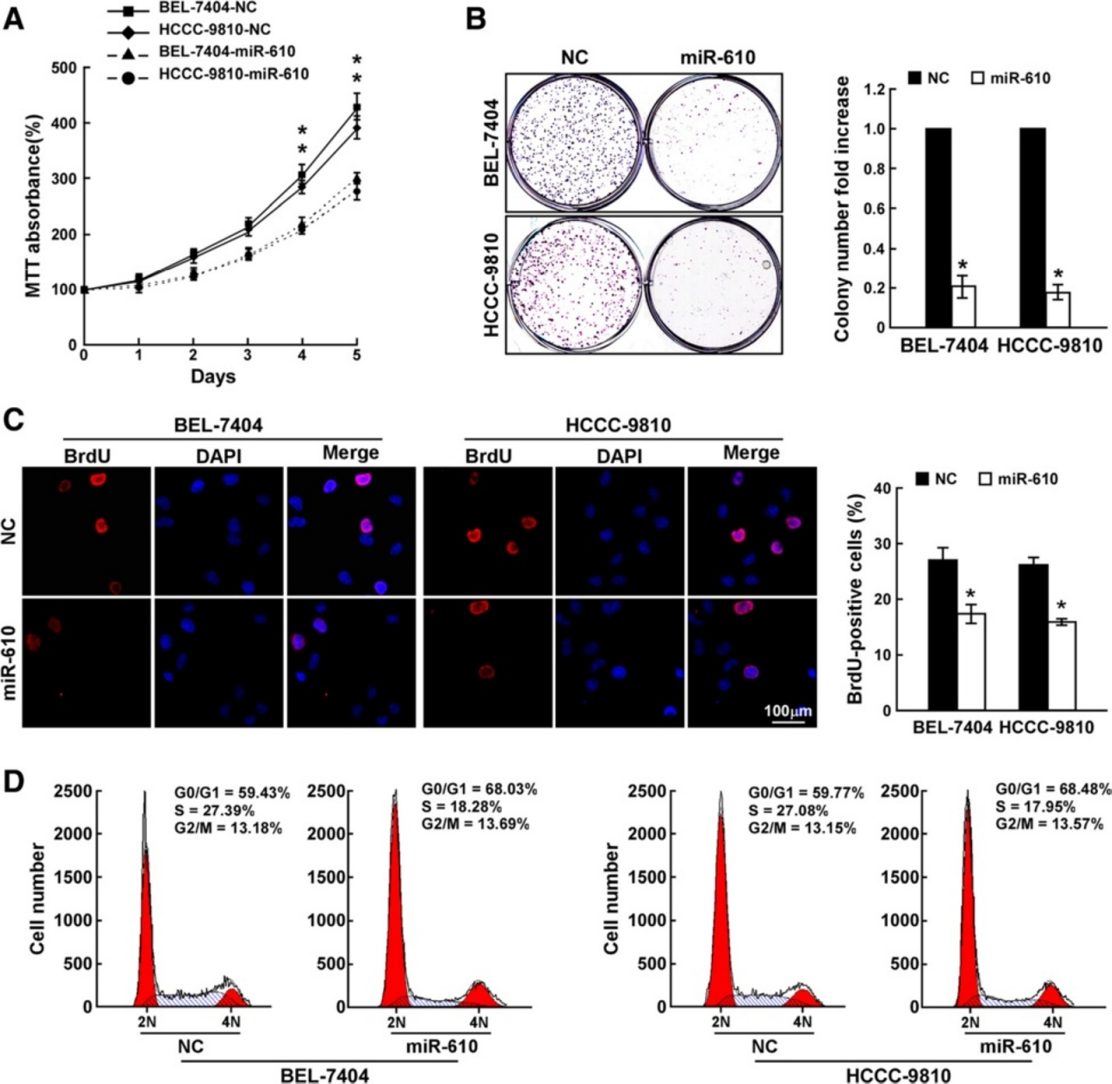
**Ectopic miR-610 expression inhibits HCC cell proliferation and cell cycle progression. A**, MTT analysis of cell growth rates of indicated cell lines after seeding. **B**, Representative micrographs (left) and quantification (right) of HCC cell colonies determined by colony formation assay. **C**, Representative micrographs (left) and quantification (right) of BrdU incorporation in HCC cells. **D**, Flow cytometry analysis of cell cycle progression in HCC cells. Bars represent the means ± SD of three independent experiments. *P <0.05. NC, negative control.

We then determined whether miR-610 played tumor suppressive role in HCC progression. As shown in Figure 3A, cell viability, measured by MTT assay, was significantly decreased in both miR-610-overexpressing HCC cell lines. The colony formation assay revealed that ectopic miR-610 significantly reduced the colony formation rate compared with that in control cells (Figure 3B). Furthermore, DNA synthesis levels, as examined with the BrdU incorporation assay, were significantly decreased in miR-610-overexpressing HCC cells, whereas control cells had relatively higher BrdU incorporation rates (Figure 3C). Moreover, flow cytometry assay showed that miR-610-overexpressing HCC cells exhibited significantly increased percentage of G1/G0-phase cells and decreased percentage of S-phase cells (Figure 3D). Taken together, these results suggest that miR-610 suppresses HCC cell proliferation and cell cycle progression, which further supports the notion that miR-610 might be a tumor-suppressive miRNA.

### MiR-610 suppresses HCC cell tumorigenicity both *in vitro*and *in vivo*

**Figure 4:**
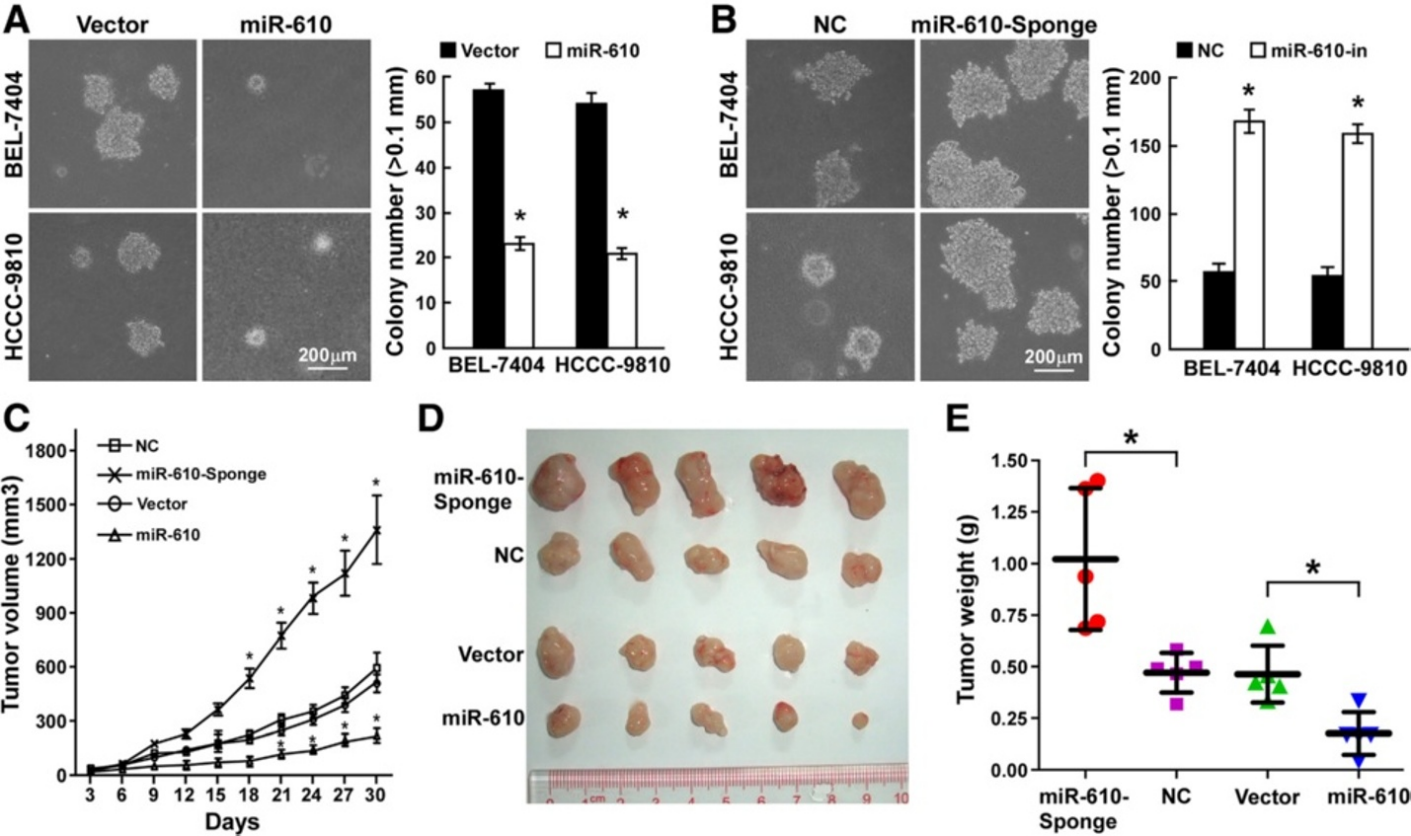
**MiR-610 suppresses HCC cell tumorigenicity both** ***in vitro*** **and** ***in vivo*** **. A** and **B**, Representative micrographs (left) and quantification (right) of colony formation determined by anchorage-independent growth assay *in vitro*. Colonies >0.1 mm were scored. **C**, Average tumor volumes (mm^3^) of different treatment groups after inoculation. **D**, Representative images of tumors in the different treatment groups. **E**, Analysis of weight of subcutaneous tumors from the different treatment groups. Bars represent the means ± SD of three independent experiments. *P <0.05. NC, negative control.

To examine the effect of miR-610 on HCC cell tumorigenicity, we established HCC cell lines, which stably expressed or inhibited miR-610, and performed an anchorage-independent growth assay (Additional file 4: Figure S3, Additional file 5: Figure S4A-B). The result showed that the HCC cells stably expressing miR-610 formed fewer and smaller colonies than the control cells did, while miR-610 inhibition using a stable miRNA sponge led to formation of more and larger colonies. We further examined miR-610 biofunction in suppression of HCC cell tumorigenicity *in vivo* by inoculating nude mice with tumor cells. The volume and weight of miR-610–overexpressing tumors were markedly lower compared with the tumors formed by control cells, indicating the suppressive function of miR-610 on HCC cell tumorigenicity *in vivo* (Figure 4C–E). Consistently, inhibition of miR-610, using the stable miRNA sponge strategy, drastically enhanced HCC cell tumorigenicity *in vivo*, for which tumor sizes were much larger (Figure 4C–E). Our results demonstrate that miR-610 could suppress HCC cell tumorigenicity both *in vitro* and *in vivo*.

### LRP6 and TBL1X are functionally relevant downstream targets of miR-610

**Figure 5:**
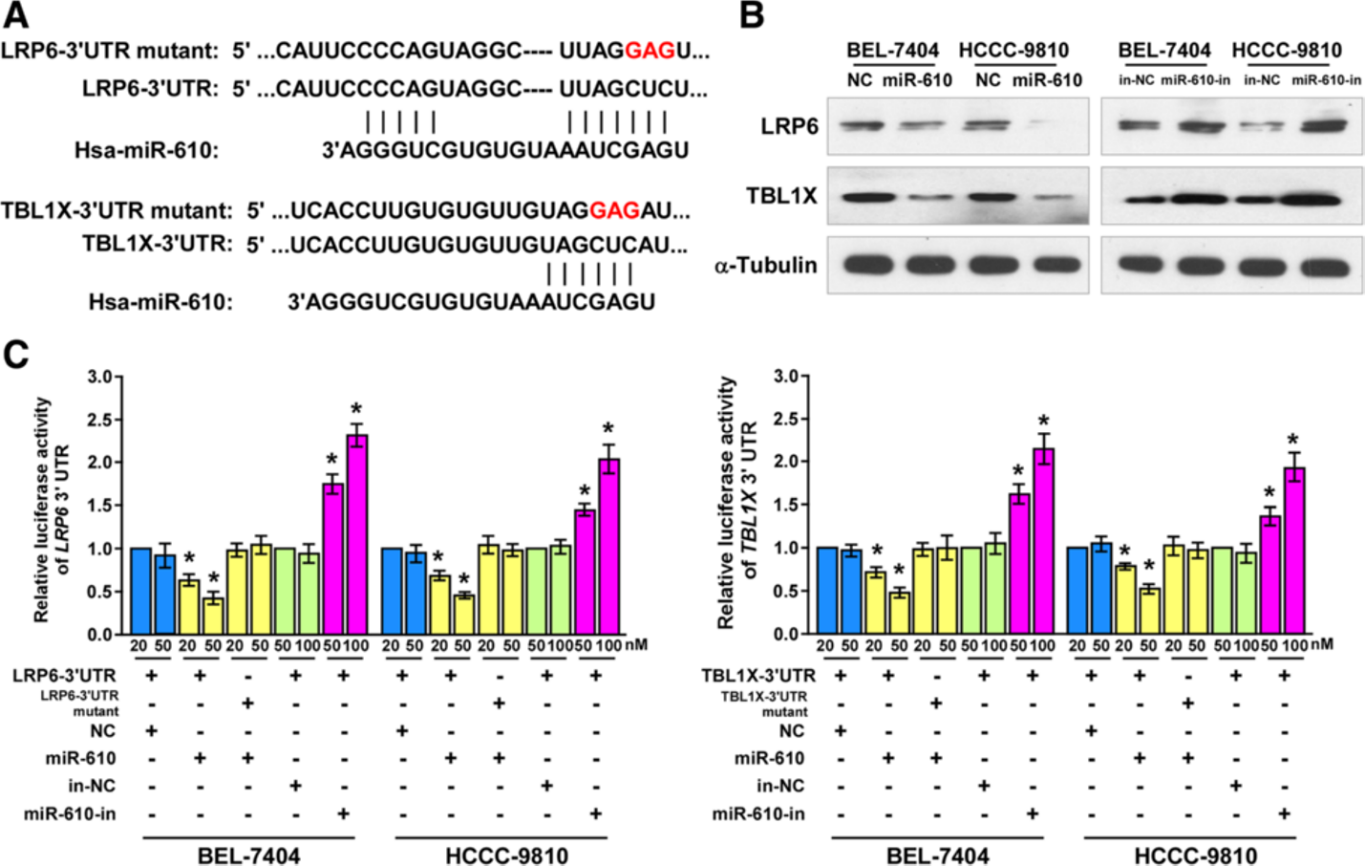
**LRP6 and TBL1X are direct targets of miR-610. A**, Schematic representation of mature miR-610 sequence and miR-610 target sites in the 3’ UTRs of the *LRP6* and *TBL1X* mRNAs. **B**, Western blot analysis of LRP6 and TBL1X expression in HCC cells transfected with miR-610 mimic, inhibitor (miR-610-in) or negative control (NC); α-tubulin was used as the loading control. **C**, Luciferase assay of pGL3-*LRP6*-3’UTR or pGL3-*TBL1X*-3’UTR reporter cotransfected with miR-610 mimic or inhibitor in HCC cells. Bars represent the means ± SD of three independent experiments. *P <0.05. Hsa, *Homo sapiens*.

The TargetScan algorithm indicated that LRP6 and TBL1X, which might be targets of miR-610 (Figure 5A). Western blotting analysis revealed that LRP6 and TBL1X expression were significant downregulated in miR-610-overexpressing cells but upregulated in miR-610-inhibited cells, while we did not observed significant alterations of LRP6 and TBL1X transcripts (Figure 5B, Additional file 5: Figure S4). To confirm whether LRP6 and TBL1X were direct targets of miR-610, luciferase reporter vector containing the 3’ UTR fragment of the *LRP6* or *TBL1X* gene was co-transfected with either miR-610 mimic or miR-610 inhibitor into HCC cells. As shown in Figure 5C, ectopic expression of miR-610 decreased, while inhibition of miR-610 increased, the luciferase activity of either the *LRP6* or *TBL1X* 3’ UTR, but not the *LRP6* or *TBL1X* 3’ UTR with mutant binding cites. Moreover, the miR-610 mutant also failed to show an inhibitory effect on the luciferase expression of either the *LRP6* or *TBL1X* 3’ UTR (Additional file 6: Figure S5A and B). Moreover, miRNPs immunoprecipitation assay revealed a selective association of miR-610 with LRP6 or TBL1X, but not with GAPDH (Additional file 6: Figure S5C and D), indicating that miR-610 negatively regulated these proteins via directly binding to their 3’UTRs. These results suggest that LRP6 and TBL1X are direct downstream targets of miR-610.

### Downregulation of miR-610 activates Wnt/β-catenin signaling

**Figure 6:**
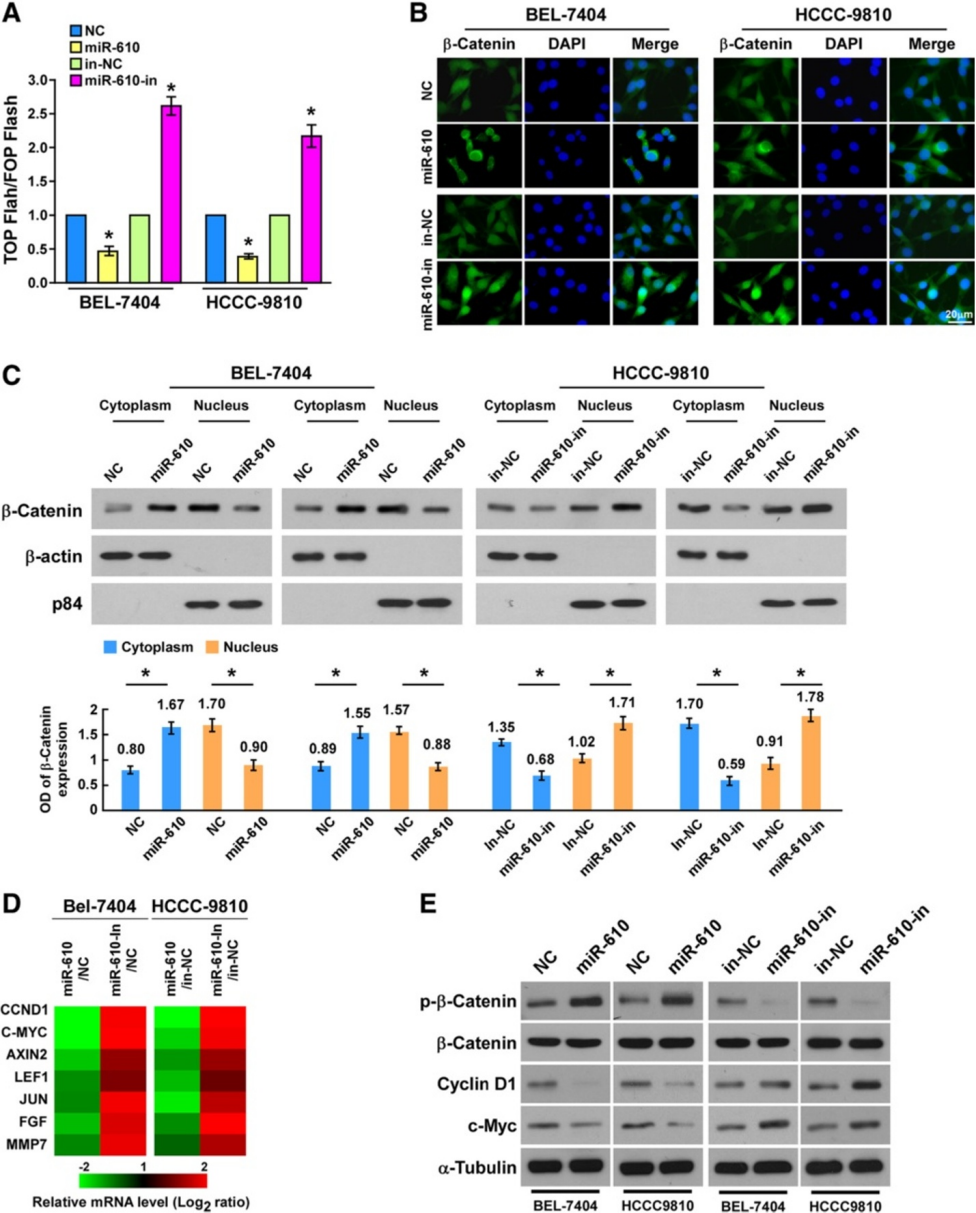
**MiR-610 suppresses β-catenin signaling pathway activity. A**, Luciferase assay of TCF/LEF transcriptional activity in indicated cells transfected with miR-610 mimic or inhibitor (miR-610-in). **B**, The cellular location of β-catenin in indicated HCC cells, as determined by immunofluorescence staining (with magnification × 1000). **C**, The expression (upper) and quantification (lower) of cytoplasm and nuclear β-catenin in indicated HCC cell lines, determined by Western blot; β-actin was used as the cytoplasm protein marker, and p84 as the nuclear protein marker. **D**, Real-time PCR analysis of mRNA expression of *CCND1*, *MYC*, *AXIN2*, *LEF1*, *JUN* and *FGF4* genes in HCC cells. **E**, Western blot measurement of phosphorylated β-catenin (p-β-catenin), β-catenin, CCND1 and c-Myc expression in HCC cells; α-tubulin was used as the loading control. Bars represent the means ± SD of three independent experiments. *P <0.05. NC, negative control.

As both TBL1X and LRP6 were demonstrated to play important roles in activation of Wnt/β-catenin pathway [18, 19, 24, 25], therefore, we further examined whether miR-610 was involved in the regulation of Wnt/β-catenin signaling. As shown in Figure 6A, the β-catenin reporter assay revealed that ectopic miR-610 remarkably decreased, while miR-610 inhibitor increased, the TCF/LEF activities in both BEL-7404 and HCCC-9810 cells. Consistently, immunofluorescence staining showed ectopic miR-610 led to significant cytoplasm location of β-catenin, whereas inhibition of miR-610 resulted in stronger nuclear signals of β-catenin (Figure 6B). In addition, the subcellular fractionation assay showed a significant nuclear accumulation of β-catenin in miR-610-inhibited BEL-7404 and HCCC-9810 HCC cell lines, whereas miR-610-overexpressed HCC cells displayed dramatically decreased β-catenin in nucleus (Figure 6C). Meanwhile, we found that the downstream target genes of Wnt/β-catenin signaling, including *CCND1*, *MYC*, *AXIN2*, *LEF1*, *JUN*, *FGF4* and *MMP7*, were downregulated in cells transfected with miR-610 mimic and upregulated in cells transfected with miR-610 inhibitor (Figure 6D). Moreover, western blotting revealed that expression levels of phosphorylated β-catenin was dramatically increased in miR-610-overexpressing cells and decreased in miR-610-inhibited cells. Consistently, the expression of Cyclin D1 and c-Myc were suppressed by ectopic miR-610 expression and upregulated by miR-610 inhibition (Figure 6E). Taken together, these data suggest that downregulation of miR-610 activates Wnt/β-catenin signaling and promotes TCF/LEF transcriptional activity.

Consistently, we also found that inhibition of miR-610 function by miR-610-sponge suppressed the expression of LRP6 and TBL1X, accompanied with the high level of nuclear expression of β-catenin, in the xenograft tumors. However, overexoressing miR-610 resulted in the upregulation of LRP6 and TBL1X and reduced nuclear β-catenin in the xenograft tumors (Additional file 7: Figure S6). These data further confirm that miR-610 suppresses HCC cell tumorigenicity by inhibit the activity of β-catenin via targeting LRP6 and TBL1X.

### Wnt/β-catenin signaling mediates miR-610-mediated HCC proliferation

**Figure 7:**
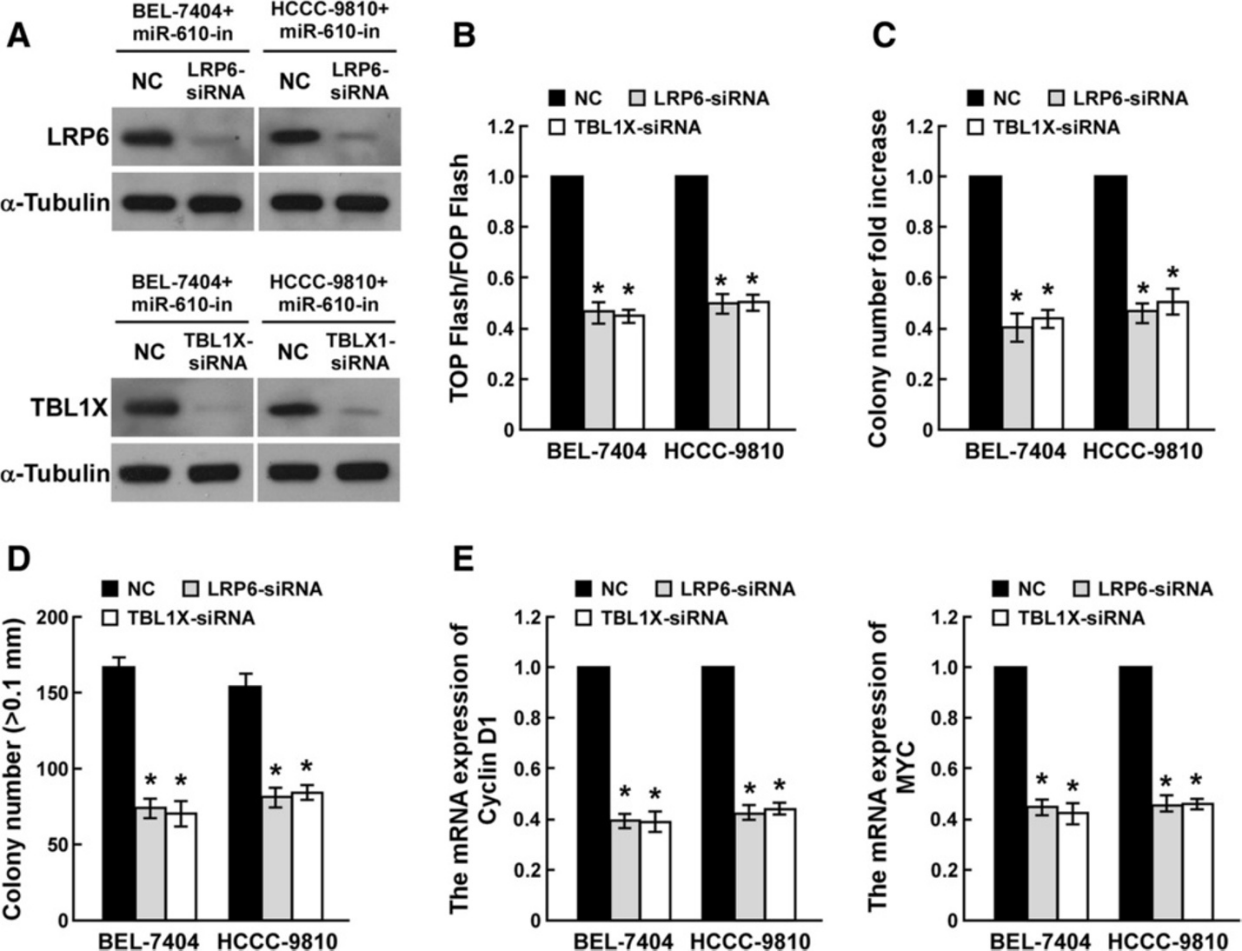
**Wnt/β-catenin signaling mediated miR-610-mediated HCC proliferation. A**, Western blot analysis of LRP6 and TBL1X expression in miR-610-inhibited HCC cells transfected with LRP6-siRNA or TBL1X-siRNA. **B**, Luciferase assay of TCF/LEF transcriptional activity in indicated cells. **C**, Quantification of indicated HCC cell colonies determined by colony formation assay. **D**, Quantification of colony formation determined by anchorage-independent growth assay. **E**, Real-time PCR analysis of mRNA expression of *CCND1* and *MYC.* Bars represent the means ± SD of three independent experiments. *P <0.05.

Next, the critical role of LRP6 and TBL1X in miR-610-inhibiting HCC cell proliferation was examined. As shown in Figure 7A-B, individual silencing of LRP6 or TBL1X potently inhibited TCF/LEF activity in miR-610 silenced cells, demonstrating that LRP6 and TBL1X are functional effectors of reduced miR-610-regulated Wnt/β-catenin activation. Inhibition of Wnt/β-catenin signaling by silencing LRP6 or TBL1X prevented the promoted miR-610-inhibitor-induced proliferative rates and the anchorage-independent growth ability (Figure 7C and D), as well as the expression of Cyclin D1 and c-Myc (Figure 7E), suggesting that LRP6 and TBL1X involved in miR-610 inhibiton-induced proliferation and tumorigenicity of HCC cells.

Furthermore, the critical role of Wnt/β-catenin signaling in miR-610-mediated HCC cell proliferation was explored. We analyzed the growth rate of miR-610-inhibited HCC cells in which the Wnt/β-catenin signaling was suppressed with TCF4-siRNA or LEF1-siRNA (Additional file 8: Figure S7A and B). Consistent with silencing LRP6 or TBL1X, inhibition of Wnt/β-catenin signaling by silencing TCF4 or LEF1, also prevented the expression of Cyclin D1 and c-Myc, the silenced miR-610-induced proliferative rates and the anchorage-independent growth ability (Additional file 8: Figure S7C-E), further suggesting that Wnt/β-catenin signaling played important role in the bio-function of miR-610 in HCC cells.

### Clinical relevance of miR-610 reduction-mediates Wnt/β-catenin activation in HCC

**Figure 8:**
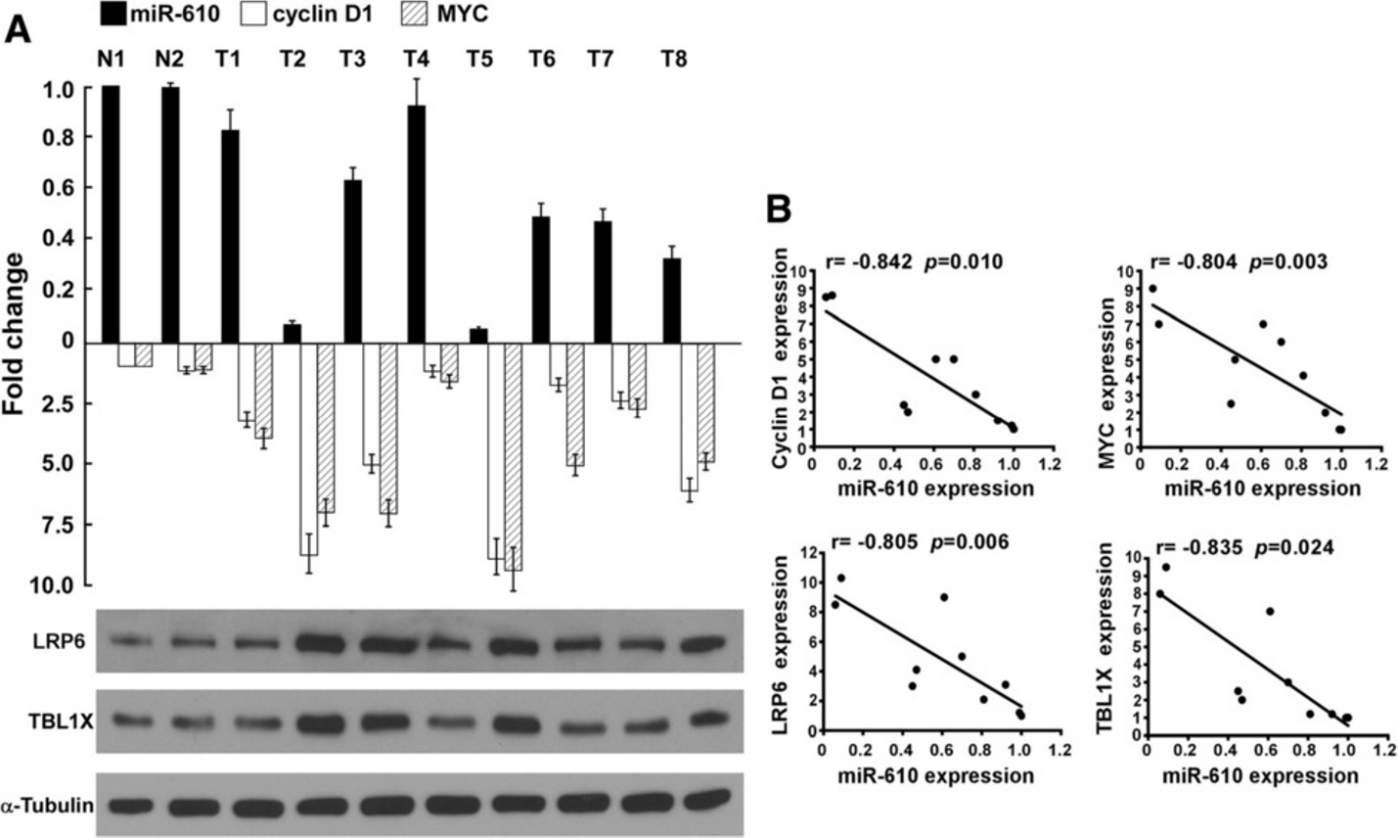
**The expression of miR-610, and Wnt/β-catenin signaling related genes in HCC tissues. A**, Real-time PCR analysis of *miR-610, Cyclin D1, MYC*, and Western blot analysis of LRP6 and TBL1X expression in HCC tissues*.* **B**, The correlation between miR-*610* expression and LRP6 and TBL1X, or *Cyclin D1* and *MYC* expression in HCC tissues. Error bars represent the mean ± SD from three independent experiments.

Finally, we examined whether miR-610 downregulation-mediated Wnt/β-catenin signaling activation in HCC cells was clinically relevant. As shown in Figure 8, miR-610 expression in 10 freshly collected HCC samples was inversely correlated with the mRNA levels of Wnt/β-catenin downstream targets, including *Cyclin D1* (r = -0.842, *P* = 0.01), *MYC* (r = -0.804, *P* = 0.03), as well as expression of *LRP6* (r = -0.805, *P* = 0.006) and TBL1X (r = -0.835, *P* = 0.024). Collectively, our results demonstrate that miR-610 downregulation activates Wnt/β-catenin signaling, resulting in HCC tumorigenicity and poorer clinical outcomes.

## Discussion

More than 80% of all HCC cases occur in developing countries, and approximately 55% of all cases occur in China, particularly in the southeast regions [38]. Therefore, discovery of effective diagnostic biomarkers and therapeutic methods is urgent. An increasing number of studies have shown that miRNAs may be significant diagnostic and prognostic markers [6, 35–37]. In the current study, we found that miR-610 was downregulated in HCC tissue and reduced miR-610 levels were significantly correlated with HCC progression and poor patient survival, suggesting that reduced miR-610 might play essential roles in HCC progression and represent a potential target for HCC therapy.

Numerous-reports demonstrated that the Wnt/β-catenin signaling pathway plays important roles in the progression of various human cancer types via modulation of many biological processes, including cell growth, invasion and metastasis, apoptosis, differentiation and stem cell development [39–42]. Herein, we demonstrated that miR-610 suppressed HCC cell proliferation and tumorigenicity both *in vitro* and *in vivo* by regulating the Wnt/β-catenin signaling pathway. Previously, it has reported that multiple downstream target genes of Wnt/β-catenin pathway were increased in various malignancies, which were correlated with tumor progression and prognosis [8, 9, 14, 43]. We examined the expression of the main downstream target genes of the β-catenin signaling pathway, i.e. *CCND1*, *MYC*, *AXIN2*, *LEF1*, *JUN*, *FGF4* and *MMP7*, and found that ectopic miR-610 decreased the mRNA expression of these genes, suggesting miR-610 modulates β-catenin signaling pathway. As Wnt/β-catenin signaling pathway regulates a series of genes related to biological progression in various tumors, it would be interesting to further investigate whether miR-610 also contributes to the aggressiveness of HCC, such as invasion and metastasis.

Since cancer is a heterogeneous and multi-step disease that cannot be successfully treated by targeting a single gene of interest, therefore understanding of the regulatory networks of many molecules will aid the exploration of effective therapeutic methods. It has been reported that miR-21 is involved in glioblastoma progression and is recognized as an anti-apoptotic factor due to its ability to block the genes responsible for controlling apoptosis [44]. MiR-486 overexpression correlates with progression of gliomas and promotes glioma aggressiveness by sustaining nuclear factor κB (NF-κB) activity via disrupting multiple NF-κB negative feedback loops [45]. miRNA-374a promotes breast cancer metastasis by downregulating WIF1, PTEN and WNT5A expression, consequently activating WNT/β-catenin signaling [46]. Our study suggests that miR-610 inhibits HCC cell proliferation and tumorigenesis through direct and specific regulation of LRP6 and TBL1X, which have been demonstrated to acted as positive regulators of the β-catenin signaling pathway. The results provide more information for establishing effective and promising therapeutic strategies aiming at miRNA-modulating networks.

Both LRP6 and TBL1X are found to function as oncogenes during tumor progression [18, 23, 47]. LRP6 is found to be targeted and suppressed by miR-126-3p leading to inhibition of tumor metastasis and angiogenesis of hepatocellular carcinoma [48]. Zhang Y *et al.* found that miR-202 suppresses cell proliferation in human hepatocellular carcinoma by downregulating LRP6 protein expression [49]. Meanwhile, it has been reported that miR-483-5p modulates the protein level of TBL1X, which is one of the Methyl CpG-binding protein 2 (MeCP2)-interacting corepressor complexes during human fetal development [50]. In the current study, we found LRP6 and TBL1X are targeted by miR-610, and overexpression of miR-610 could inhibit proliferation and tumorigenesis of HCC cells by suppressing the expression of LRP6 and TBL1X, followed by downregulation of β-catenin signaling activity.

MiR-610 locates at chromosome 11p14.1. The region 11p14.1 deletion was found to be associated with multiple disease, such as Attention-Deficit Hyperactivity Disorder (ADHD), autism, developmental delay, obesity, neurobehavioral problems and WAGR syndrome (Wilms tumor, aniridia, genitourinary anomalies, and mental retardation) [51, 52]. Therefore, it would be of great interest to further investigate whether reduced miR-610 in HCC is attributed to genomic deletion and/or other transcriptional regulation mechanism.

## Conclusion

In summary, we observed that miR-610 expression was reduced in HCC cells and tissues, which was correlated with HCC progression and survival. Consistently, miR-610 overexpression inhibited HCC cell proliferation and tumorigenicity, while miR-610 inhibition enhanced cell growth and tumor formation. Furthermore, we demonstrated that miR-610 directly suppressed LRP6 and TBL1X, which resulted in activation of WNT/β-catenin signaling activity. Our findings reveal novel roles for miR-610 in HCC development and progression and suggest miR-610 as a potential target for HCC diagnosis and treatment.

## Methods

### Cell culture

The immortalized normal liver epithelial cell lines THLE-3 and LO2 were purchased from American Type Culture Collection (Manassas, VA, USA) and cultured according to the manufacturer’s instructions. HCC cell lines (HepG2, BEL-7404, Huh7, BEL-7402, PLC/PRF, Hep3B, HCCC-9810) were purchased from American Type Culture Collection and maintained in RPMI 1640 medium (Invitrogen, Carlsbad, CA, USA) supplemented with 10% fetal bovine serum (FBS, Invitrogen) at 37°C in a 5% CO_2_ incubator.

### Generation of stably engineered cell lines

The miR-610 expression plasmid pMSCV-miR-610 was generated by cloning the genomic precursor miR-610 gene into a retroviral transfer plasmid pMSCVpuro (Promega, Madison, WI, USA). pMSCV-miR-610 was then cotransfected with the packaging plasmid into 293FT cells using the standard calcium phosphate transfection method [53]. Puromycin (0.5 μg/ml, Sigma-Aldrich) was used to select stably transduced cells. Real-time quantitative polymerase chain reaction (PCR) was used to confirm miR-610 expression. MiR-610 mimic, inhibitor and negative control were purchased from RiboBio (Guangzhou, China). Oligonucleotide transfection was performed using Lipofectamine 2000 (Invitrogen) according to the manufacturer’s instructions.

### Tissue specimens and patient information

We examined 76 paraffin-embedded, archived HCC specimens and 10 pairs of snap-frozen HCC tumors and matched adjacent normal tissues that had been histopathologically diagnosed and verified by experienced pathologists. The fresh tissues were frozen and stored in liquid nitrogen until further use. Prior patient consent and approval from the Institute Research Ethics Committee were obtained for the use of clinical materials for research purposes.

### RNA extraction and real-time quantitative PCR

Total cellular RNA was extracted using TRIzol (Invitrogen) according to the manufacturer instructions. Reverse transcription was performed using the M-MLV Reverse Transcription system (Promega). Real-time PCR was performed using a standard SYBR Green PCR kit protocol (Applied Biosystems, Foster City, CA) in an ABI PRISM 7500 Sequence Detection System (Applied Biosystems). Gene expression levels were normalized to the housekeeping gene glyceraldehyde-3-phosphate dehydrogenase (*GAPDH*) as the control and calculated as 2^-[(Target gene Ct) – (*GAPDH* Ct)]^, where C_t_ represents the threshold cycle for each transcript. The relative expression levels were calculated as 2^-[(miR-610 Ct) – (U6 Ct)]^ following normalization with reference to the expression of small nuclear RNA U6.

The primers used were: LRP6 forward, 5′-TCAGTCCATTT GGCCAGTAA-3′, reverse: 5′-CAACCCAGAGCTATTGCCTT-3′; TBL1X forward, 5′-CAGGGCTCCTTATGGTG ACT -3′, reverse: 5′- CATATCAGATG CCTCGCAGA -3′;cyclin D1 (*CCND1*) forward, 5′-AACTACCTGGACCGCTTCCT-3′, reverse: 5′-CCACTTGAGCTT GTTCACCA-3′; *MYC* forward: 5′-TCAAGAGGC GAACACACAAC-3′, reverse: 5′-GGCCTTTTCATTGTTTTC CA-3′; *AXIN2* forward: 5′-TTATGCTTTGCACTACGTCC CTCCA-3′, reverse: 5′-CGCAAC ATGGTCAAC CCTCAGAC-3′; lymphoid enhancer - binding factor 1 (*LEF1*) forward: 5′-CACTGTA AGTGATGAGGGGG-3′, reverse: 5′-TGGATCTCTTTCTCCACCCA-3′; *JUN* forward: 5′-CAGGTGGCACAGCTTAAACA-3′, reverse: 5′-GTTTGCAACTGCTGCGTTA G-3′; fibroblast growth factor 4 (*FGF4*) forward: 5′-CGTGGTGAGCATCTTCGGAGTGG-3′, reverse: 5′-CCTTCTTGGTCCGCCCGTTC TTA-3′; matrix metalloproteinase 7 (*MMP7*) forward: 5′-GTATGGGACATTCCTCTGAT CC-3′, reverse:5′-CCAATGAATGAATGAATGG ATG-3′; *GAPDH* forward: 5′-GACTCAT GACCACAGTCCATGC-3′, reverse: 3′-AGAGGCAGGGATGATGTTCTG-5′. The primers used for miR-610 and U6 stem–loop reverse transcription–PCR were purchased from RiboBio.

### Western blotting

Cells were lysed in 1× sample buffer and protein concentrations were measured using Bio-Rad protein assay reagent (Bio-Rad Laboratories, Berkeley, CA, USA). Protein (20 μg) was separated by electrophoresis and transferred onto polyvinylidene difluoride membranes (Millipore, Billerica, MA, USA). The membranes were probed with polyclonal rabbit antibodies, anti-LRP6 (Cell Signaling, Danvers, MA, USA), anti-TBL1X (Sigma-Aldrich), anti-β-catenin, anti-phospho-β-catenin, anti-CCND1 and anti-c-Myc (Cell Signaling). The membranes were then stripped and re-probed; an anti-α-tubulin antibody (Cell Signaling) was used as a loading control.

### Cell viability assay

Cell viability was measured by MTT assay. Cells (1 × 10^4^) were cultured in 96-well plates and stained at the indicated time points with 100 μl sterile MTT (0.5 mg/ml; Invitrogen) for 4 h at 37°C, followed by removal of the culture medium and the addition of 150 μl dimethyl sulfoxide (Sigma-Aldrich), followed by measurement of the absorbance at 570 mm. Relative cell numbers were calculated in sextuplicate in three independent experiments.

### Colony formation assay

Cells were trypsinized and seeded in 6-well plates (1 × 10^3^ cells per well). After 10 days, cells were fixed with 10% formaldehyde for 15 min, stained with 1.0% crystal violet for 5 min, and then counted and photographed. All experiments were performed in triplicate.

### BrdU incorporation assay

The level of DNA synthesis was determined by estimating DNA uptake of 5-bromo-2’-deoxyuridine-5’-monophosphate (BrdU). Cells were trypsinized, transferred to a sterile coverslip and allowed to settle. After 48-h serum starvation and 4 h incubation in complete medium, cells were fixed and permeabilized with 0.1% Triton for 10 min. Subsequently, cells were labeled with BrdU (10 μM; Sigma-Aldrich) for 1 h, incubated in serum-free medium containing anti-BrdU antibody for 1 h at 37°C and incubated with 4’, 6-diamidino-2-phenylindole for nuclear staining. Each experiment was repeated three times independently; stained cells were counted under a fluorescence microscope (Olympus, Tokyo, Japan).

### Flow cytometry analysis

Cells were harvested by trypsinization and fixed in 80% ice-cold ethanol in phosphate-buffered saline. Bovine pancreatic RNase (2 μg/ml; Sigma-Aldrich) was added to the cells, followed by 30-min incubation at 37°C, and then 30-min incubation in propidium iodide (10 μg/ml, Invitrogen) at room temperature. Propidium iodide–stained cells (>10,000 cells) were analyzed using a FACSCalibur flow cytometer (BD Biosciences, San Jose, CA, USA). All experiments were performed in triplicate.

### Anchorage-independent growth assay

RPMI 1640 medium (1.5 ml) containing 10% FBS and 0.33% agar was plated in 6-well plates that were stored at 4°C for the agar to solidify. The cells were trypsinized and 1 × 10^3^ cells per well were mixed with RPMI 1640 medium containing 10% FBS and 0.66% agar and plated on the prepared 6-well plates. After 10-day incubation, colony sizes were measured using an ocular micrometer; colonies >0.1 mm in diameter were scored. All experiments were performed in triplicate.

### Xenograft tumors

We used 5-week-old BALB/c nude mice for the HCC xenograft model. Medium (0.2 ml) containing 5 × 10^6^ HCC cells were injected subcutaneously into the left and right posterior flank regions of each mouse. Mice were housed in a pathogen-free environment and tumor growth was examined every three days. Mice were sacrificed after 21 days, and the weight and volume of each tumor were calculated. All experimental procedures were conducted in accordance with the Guide for the Care and Use of Laboratory Animals and conformed to our institutional ethical guidelines for animal experiments.

### Luciferase assay

The 3’ UTR of the *LRP6* or *TBL1X* gene was PCR-amplified from genomic DNA and inserted downstream of the luciferase reporter gene in a pGL3 reporter vector (Promega). The primer sets used were: 3’-UTR of LRP6 containing the miR-610 binding site, 5′- AATCCGCGGGG GTTGTATTTCTTTATCATT -3′ and 5′- GCCCTGCAG CGCATACCTCTTCAGTCTC -3′; 3′-UTR of TBL1X containing the miR-610 binding site, 5′- AATCCGCGGTGTCTTGGG CTTGTTGTC -3′ and 5′- GCCCTGCATCTGTGGC TTC TTCGGTTC -3′. LRP6-3′UTR mutant: 5′- AGCTCCATTCCCCAGTAGGCTTAGGAGTTC AATTTGACT GCTGTTTTTGC-3′ and 5′- CAGCAGTCAAATTGAACTCCTAAGCCTA CTGGGGAATGGAGCT-3′; TBL1X-3’UTR mutant: 5′- ATCACCTTGTGTGTTGTAG GAGA TTTGTTTCAAGAGAGAATCAACAGATC-3′ and 5′- GATCTGTTGATTCTCT CTTGAAA CAAATCTCCTACAACACACAAGGTGAT-3′. Reporter plasmids containing wild-type (CCTTTGATC; TOPflash) or mutated (CCTTTGGCC; FOPflash) T cell factor (TCF)/LEF DNA binding sites were purchased from Upstate Biotechnology (Lake Placid, NY, USA). Cells were plated in a 24-well plate and incubated for 24 h prior to transfection. Firefly luciferase constructs containing the 3’ UTR of the potential miR-610 target, pRL-TK *Renilla* luciferase normalization control (Promega), miRNA mimic, inhibitor or negative control were cotransfected using Lipofectamine 2000 (Invitrogen). Lysates were collected 48 h after transfection and measured using a Dual-Luciferase Reporter System (Promega) according to the manufacturer protocol. Three independent experiments were performed and the data presented as the means ± SD.

### Nuclear protein extraction assay

The nuclear protein extraction assay was conducted using Nuclear Extraction Kit (Life Technologies, Carlsbad, CA, USA). Briefly, Collect cells (5 × 10^6^) were collected and washed by cold PBS, and gently resuspended with 500 μl 1× Hypotonic Buffer, followed by incubating on ice for 15 minutes. Add 25 μl detergent (10% NP40) and vortex for 10 seconds at highest setting. Centrifuge the homogenate for 10 minutes at 3,000 rpm at 4°C. Transfer and save the supernatant. This supernatant contains the cytoplasmic fraction. The pellet is the nuclear fraction. Resuspend nuclear pellet in 50 μl complete Cell Extraction Buffer for 30 minutes on ice with vortexing at 10 minute intervals. Centrifuge for 30 minutes at 14,000 g at 4°C. Transfer supernatant (nuclear fraction) to a clean microcentrifuge tube. The nuclear extracts are ready for assay.

### Micro-ribonucleoprotein complex immunoprecipitation assay

Cells were cotransfected with a plasmid encoding hemagglutinin–argonaute 1 (HA-Ago1 or -Ago2) (Addgene, Cambridge, MA, USA) and miR-610 mimic (100 nM), followed by HA-Ago1 or -Ago2 immunoprecipitation (IP) using an anti-HA antibody (Roche Applied Science, Mannheim, Germany). The association of the *LRP6* and *TBL1X* mRNA with the RNA-induced silencing complex was tested using real-time PCR analysis of the IP product. The primers used: LRP6, 5′- CGACTTGAA CCATCCATTCC-3′ and 5′- CAACCCAGAGCTATTGCCTT-3′; TBL1X, 5′- TGTATG GACCTGTGGACCAG-3′ and 5′- CATATCAGATGCCTCGCAGA-3′.

### Statistical analysis

All statistical analyses were performed using SPSS software. The association between miRNA expression and tumor stage was assessed using the Fisher exact test or Pearson *χ*^2^ test. We used the Kaplan–Meier method to estimate survival; log-rank testing was used to test differences between survival curves. Data are reported as means ± SD; mean values were compared using Student’s *t*-test. Results were considered statistically significant when *P* <0.05.

## Authors’ information

Xian-Cheng Zeng: Department of Hepatic Surgery, The Third Affiliated Hospital of Sun Yat-sen University, Department of General Surgery, Zengcheng People’s Hospital, (BoJi-Affiliated Hospital of Sun Yat-Sen University), Department of clinical laboratory, Zengcheng People’s Hospital (BoJi-Affiliated Hospital of Sun Yat-Sen University), Department of Hepatic Surgery, The Third Affiliated Hospital of Sun Yat-sen University, Guangzhou, Guangdong, China; Fo-Qiu Liu: Department of Gastroenterology, Zengcheng People,s Hospital (BoJi-Affiliated Hospital of Sun Yat-Sen University), Zengcheng, Guangdong, China; Rong Yan: Department of Hepatic Surgery, The Third Affiliated Hospital of Sun Yat-sen University, Guangzhou, Guangdong, China; Hui-Min Yi: Department of Hepatic Surgery, the Third Affiliated Hospital of Sun Yat-sen University; Tong Zhang: Department of Hepatic Surgery, the Third Affiliated Hospital of Sun Yat-sen University; Guo-Ying Wang: Department of Hepatic Surgery, the Third Affiliated Hospital of Sun Yat-sen University; Yang Li: Department of Hepatic Surgery, the Third Affiliated Hospital of Sun Yat-sen University; Nan Jiang: Department of Hepatic Surgery, the Third Affiliated Hospital of Sun Yat-sen University.

## Electronic supplementary material

Additional file 1: Figure S1: Analysis of miR-610 expression in a published, microarray-based high-throughput assessment (NCBI/GEO/GSE31384, n =166, *P* <0.05). (TIFF 224 KB)

Additional file 2: Table S1: Clinicopathological characteristics of studied patients and expression of miR-610 in HCC. **Table S2.** Correlation between the clinicopathological features and expression of miR-610. (DOC 89 KB)

Additional file 3: Figure S2: Ectopic miR-610 expression inhibits HCC cell HepG2 and Hep3B proliferation and colony formation. **A**, MTT analysis of cell growth rates of indicated cell lines after seeding. **B**, Representative micrographs (left) and quantification (right) of HCC cell colonies determined by colony formation assay. Bars represent the means ± SD of three independent experiments. *P <0.05. NC, negative control. (TIFF 491 KB)

Additional file 4: Figure S3: The expression of miR-610 in xenograft tumors, determined by Real-time PCR. Average miR-610 expression was normalized using U6 expression. Bars represent the means ± SD of three independent experiments. *P <0.05. (TIFF 31 KB)

Additional file 5: Figure S4: The expression of LRP6 and TBL1X mRNA in indicated cells, determined by Real-time PCR. Average mRNA expression was normalized using GAPDH expression. Bars represent the means ± SD of three independent experiments. *P* >0.05. (TIFF 177 KB)

Additional file 6: Figure S5: LRP6 and TBL1X are direct targets of miR-610. **A**, Schematic representation of mutant miR-610 sequence and miR-610 target sites in the 3’ UTRs of the *LRP6* and *TBL1X* mRNAs. **B**, Luciferase assay of pGL3-*LRP6*-3’UTR or pGL3-*TBL1X*-3’UTR reporter cotransfected with miR-610 mimic or miR-610-mut in HCC cells. **C** and **D**, MiRNP IP assay revealing the association between miR-610 and LRP6 or TBL1X. MiRNP IP assay was conducted using AGO1 **(C)** and AGO2 **(D)** plasmid. Bars represent the means ± SD of three independent experiments. *P <0.05. (TIFF 314 KB)

Additional file 7: Figure S6: The expression of LRP6, TBL1X and β-catenin in xenograft tumors, determined by immunohistochemistry. (TIFF 2 MB)

Additional file 8: Figure S7: Wnt/β-catenin signaling mediated miR-610-mediated HCC proliferation. **(A)** Western blot analysis of TCF4 and LEF1 expression in miR-610-inhibited HCC cells transfected with TCF4-siRNA or LEF1-siRNA. **(B)** Luciferase assay of TCF/LEF transcriptional activity in indicated cells. **(C)** Real-time PCR analysis of mRNA expression of *CCND1* and *MYC.* **(D)** Quantification of indicated HCC cell colonies determined by colony formation assay. **(E)** Quantification of colony formation determined by anchorage-independent growth assay. Bars represent the means ± SD of three independent experiments. *P <0.05. (TIFF 439 KB)

## Abbreviations

LRP6: Lipoprotein receptor-related protein 6
TBL1X: Transducin β–like protein 1
HCC: Hepatocellular carcinoma
miRNA or miR: microRNAs
3′UTR: 3′untranslated region
NF-κB: Nuclear factor κB
BrdU: 5-bromo-2′-deoxyuridine-5′-monophosphate
MTT: 3-[4, 5-dimethylthiazol-2-yl]-2, 5-diphenyltetrazolium bromide.
