# Supplementary figures and images for: Downregulation of miR-610 promotes proliferation and tumorigenicity and activates Wnt/β-catenin signaling in human hepatocellular carcinoma
Source: Mol Cancer. 2014 Dec 10;13:261. doi: 10.1186/1476-4598-13-261 (PMC4295306; doi:10.1186/1476-4598-13-261)

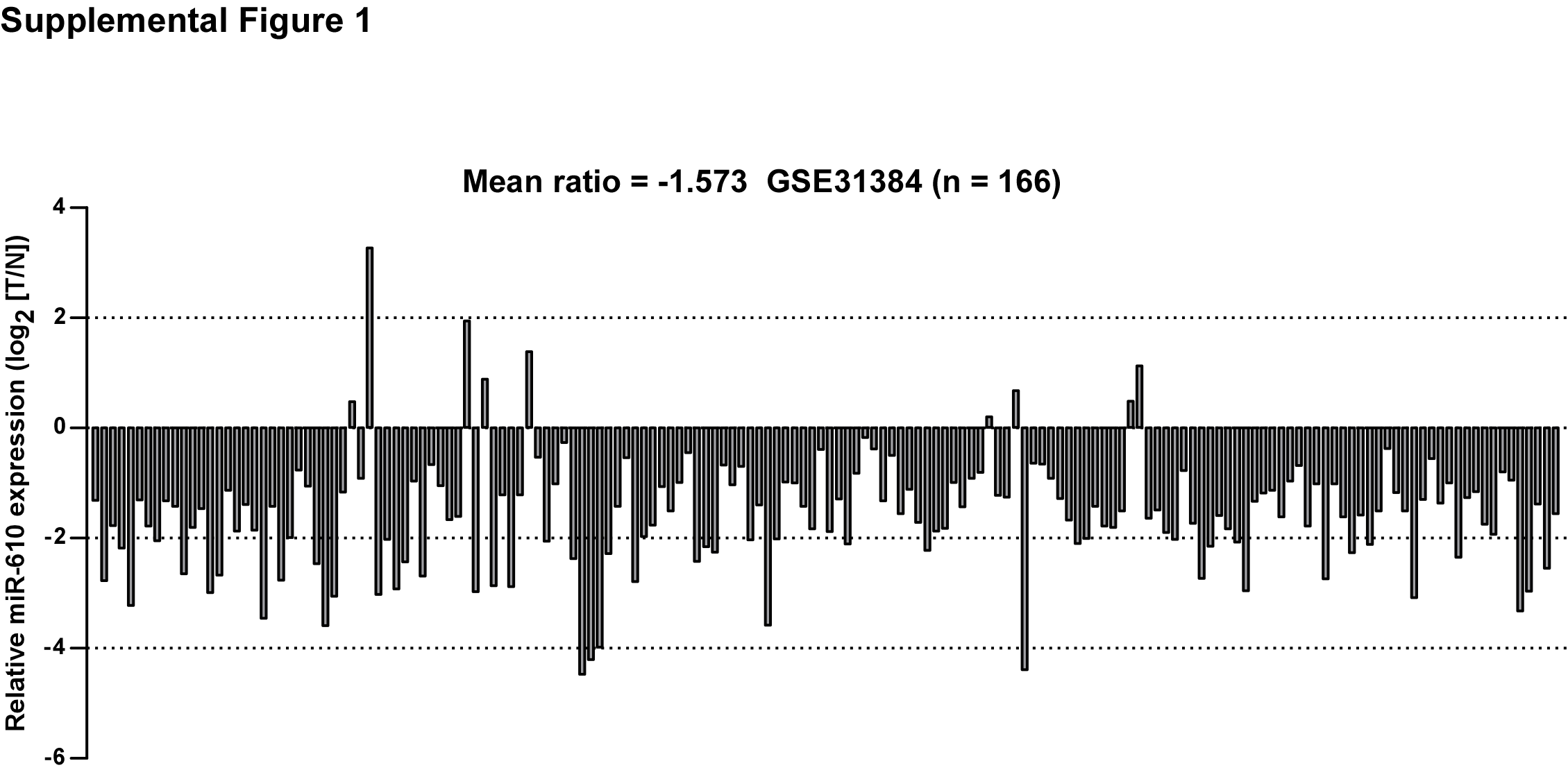

Supplement: Supplementary file 1 — Additional file 1: Figure S1: Analysis of miR-610 expression in a published, microarray-based high-throughput assessment (NCBI/GEO/GSE31384, n =166, P <0.05). (TIFF 224 KB) [file 12943_2014_1458_MOESM1_ESM.tiff]

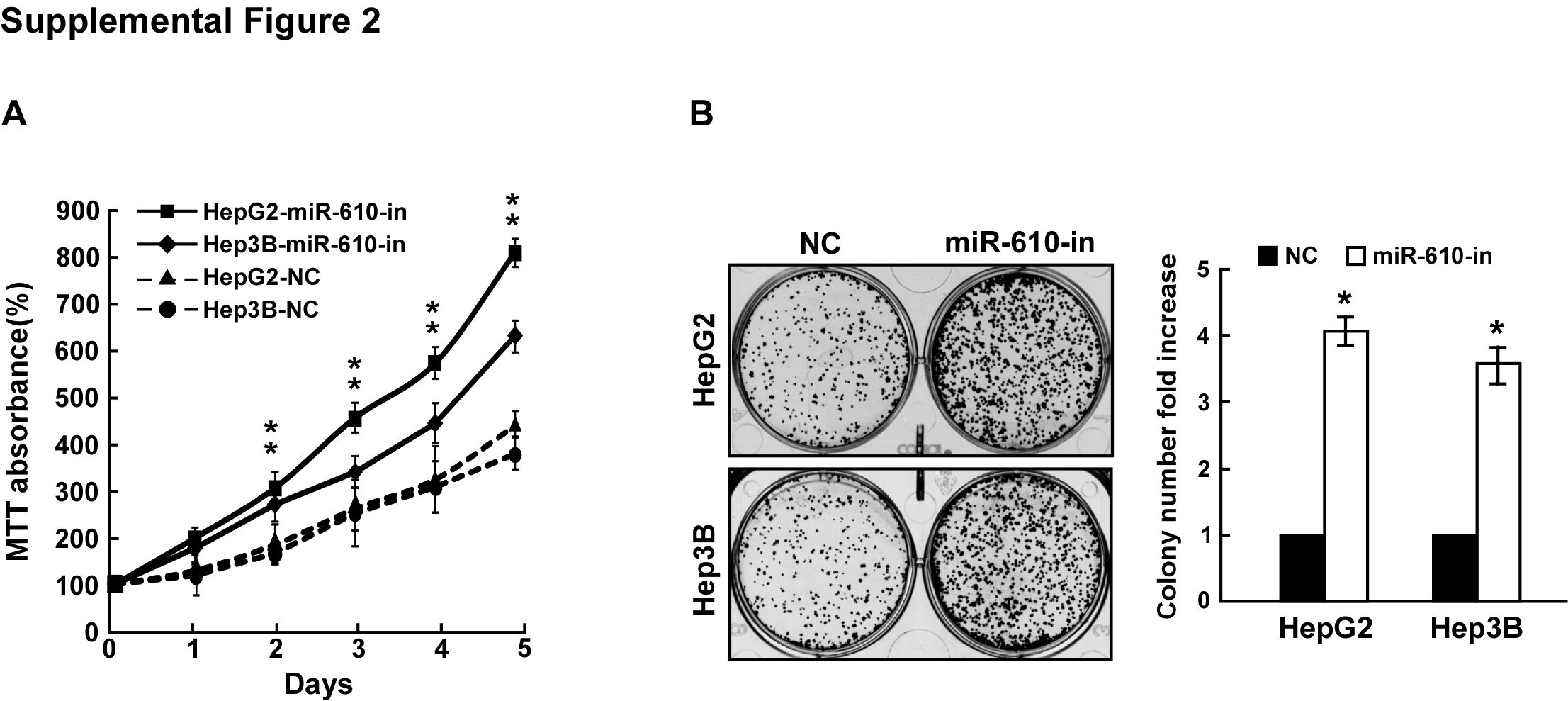

Supplement: Supplementary file 3 — Additional file 3: Figure S2: Ectopic miR-610 expression inhibits HCC cell HepG2 and Hep3B proliferation and colony formation. A, MTT analysis of cell growth rates of indicated cell lines after seeding. B, Representative micrographs (left) and quantification (right) of HCC cell colonies determined by colony formation assay. Bars represent the means ± SD of three independent experiments. *P <0.05. NC, negative control. (TIFF 491 KB) [file 12943_2014_1458_MOESM3_ESM.tiff]

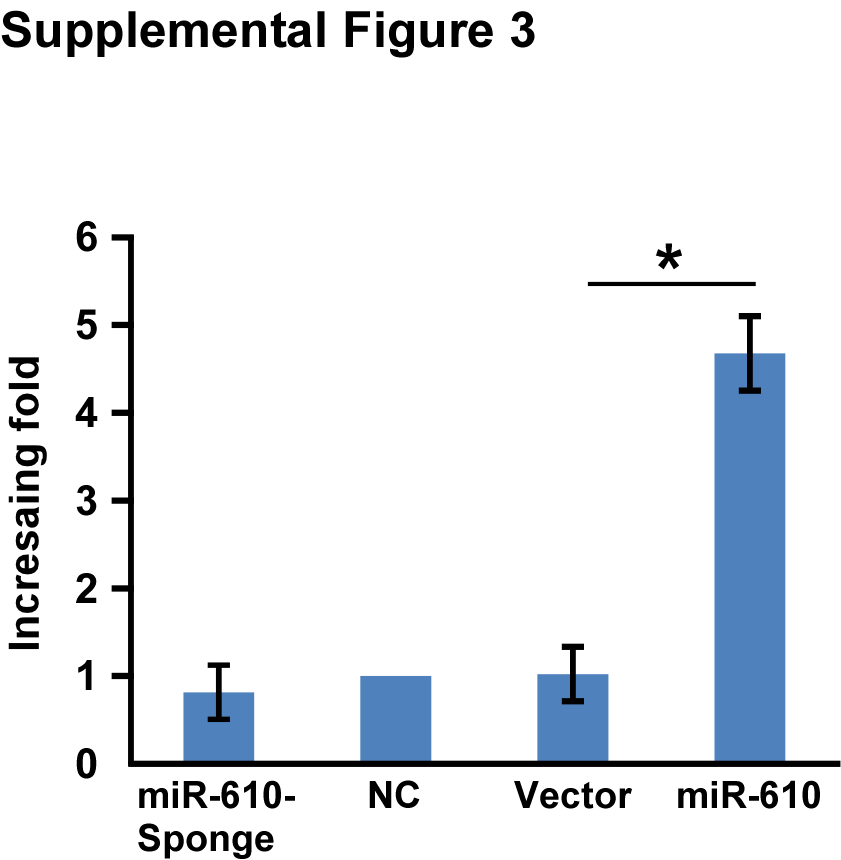

Supplement: Supplementary file 4 — Additional file 4: Figure S3: The expression of miR-610 in xenograft tumors, determined by Real-time PCR. Average miR-610 expression was normalized using U6 expression. Bars represent the means ± SD of three independent experiments. *P <0.05. (TIFF 31 KB) [file 12943_2014_1458_MOESM4_ESM.tiff]

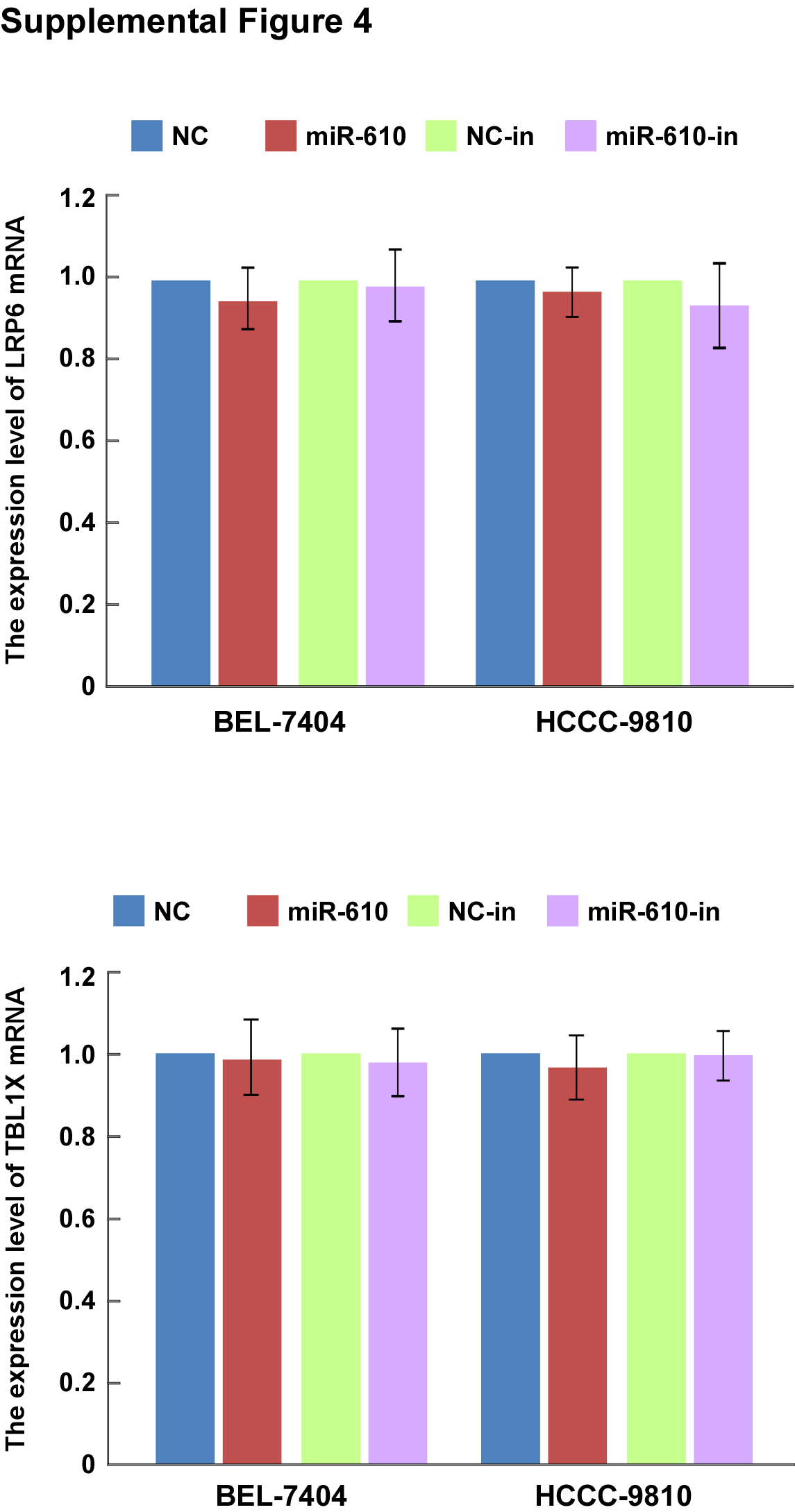

Supplement: Supplementary file 5 — Additional file 5: Figure S4: The expression of LRP6 and TBL1X mRNA in indicated cells, determined by Real-time PCR. Average mRNA expression was normalized using GAPDH expression. Bars represent the means ± SD of three independent experiments. P >0.05. (TIFF 177 KB) [file 12943_2014_1458_MOESM5_ESM.tiff]

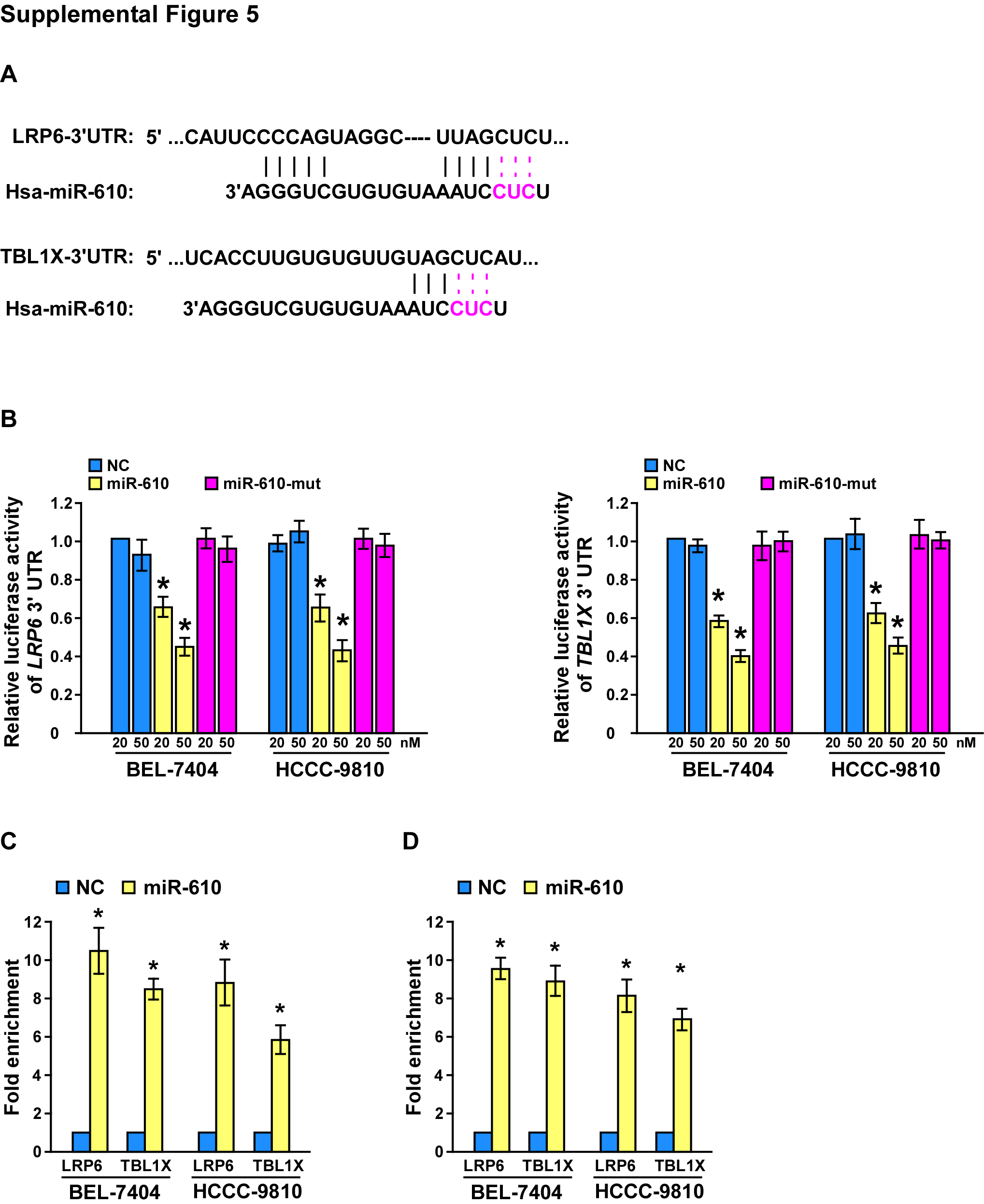

Supplement: Supplementary file 6 — Additional file 6: Figure S5: LRP6 and TBL1X are direct targets of miR-610. A, Schematic representation of mutant miR-610 sequence and miR-610 target sites in the 3’ UTRs of the LRP6 and TBL1X mRNAs. B, Luciferase assay of pGL3-LRP6-3’UTR or pGL3-TBL1X-3’UTR reporter cotransfected with miR-610 mimic or miR-610-mut in HCC cells. C and D, MiRNP IP assay revealing the association between miR-610 and LRP6 or TBL1X. MiRNP IP assay was conducted using AGO1 (C) and AGO2 (D) plasmid. Bars represent the means ± SD of three independent experiments. *P <0.05. (TIFF 314 KB) [file 12943_2014_1458_MOESM6_ESM.tiff]

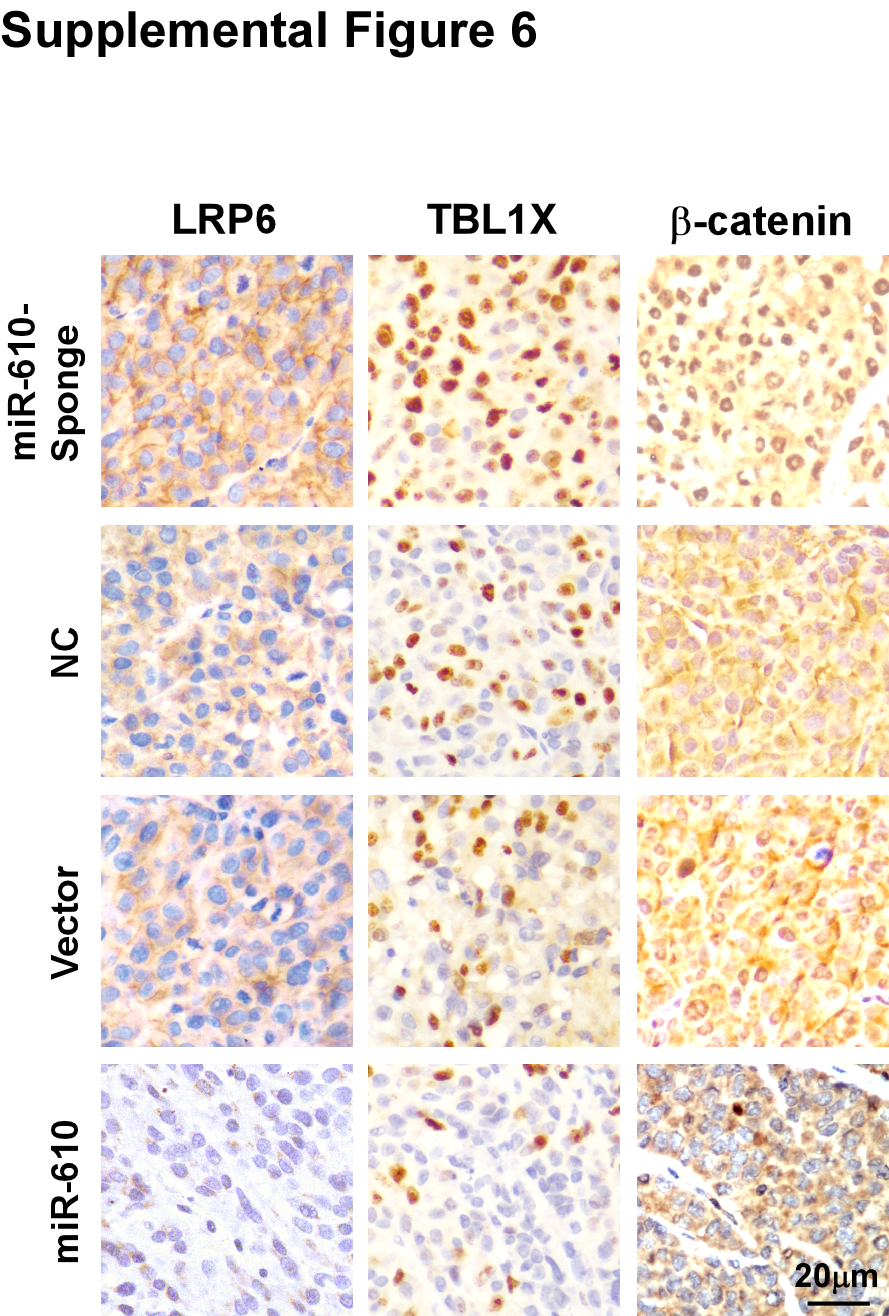

Supplement: Supplementary file 7 — Additional file 7: Figure S6: The expression of LRP6, TBL1X and β-catenin in xenograft tumors, determined by immunohistochemistry. (TIFF 2 MB) [file 12943_2014_1458_MOESM7_ESM.tiff]

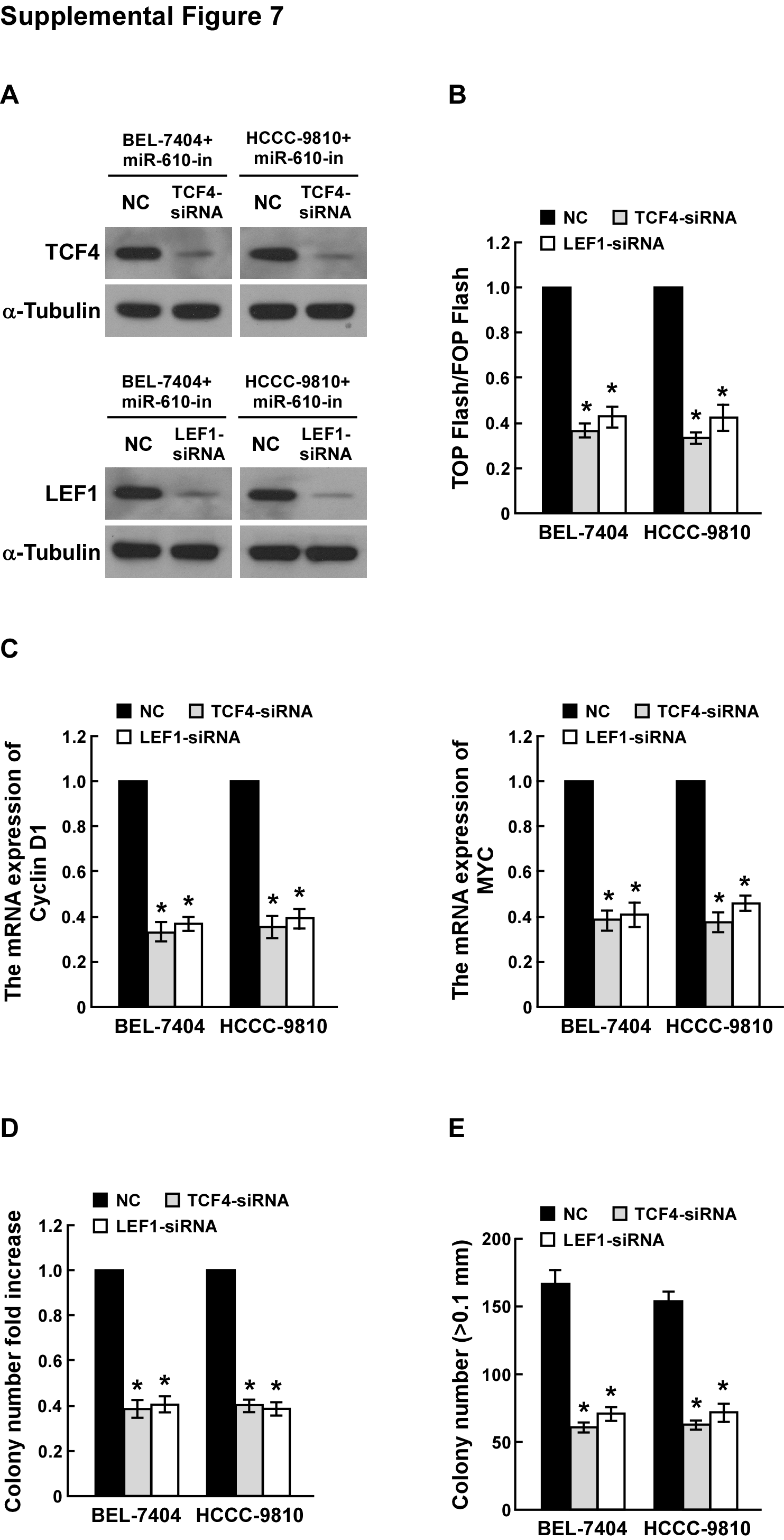

Supplement: Supplementary file 8 — Additional file 8: Figure S7: Wnt/β-catenin signaling mediated miR-610-mediated HCC proliferation. (A) Western blot analysis of TCF4 and LEF1 expression in miR-610-inhibited HCC cells transfected with TCF4-siRNA or LEF1-siRNA. (B) Luciferase assay of TCF/LEF transcriptional activity in indicated cells. (C) Real-time PCR analysis of mRNA expression of CCND1 and MYC. (D) Quantification of indicated HCC cell colonies determined by colony formation assay. (E) Quantification of colony formation determined by anchorage-independent growth assay. Bars represent the means ± SD of three independent experiments. *P <0.05. (TIFF 439 KB) [file 12943_2014_1458_MOESM8_ESM.tiff]
