# Supplementary material for: Downregulation of miR-610 promotes proliferation and tumorigenicity and activates Wnt/β-catenin signaling in human hepatocellular carcinoma
Source: Mol Cancer. 2014 Dec 10;13:261. doi: 10.1186/1476-4598-13-261 (PMC4295306; doi:10.1186/1476-4598-13-261)
Supplement: Supplementary file 2 — Additional file 2: Table S1: Clinicopathological characteristics of studied patients and expression of miR-610 in HCC. Table S2. Correlation between the clinicopathological features and expression of miR-610. (DOC 89 KB) [file 12943_2014_1458_MOESM2_ESM.doc]

**Supplementary Table 1**. Clinicopathological characteristics of studied patients and expression of miR-610 in HCC

| **Factor** | **No.** | **(%)** |
| --- | --- | --- |
| **Age (years)** |  |  |
| ≤45 | 31 | 40.8 |
| >45 | 45 | 59.2 |
| **Gender** |  |  |
| Male | 67 | 88.2 |
| Female | 9 | 11.8 |
| **Clinical stage** |  |  |
| I | 6 | 7.9 |
| II | 33 | 43.4 |
| III | 23 | 30.3 |
| IV | 14 | 18.4 |
| **T** |  |  |
| T1 | 5 | 6.6 |
| T2 | 31 | 40.8 |
| T3  T | 27 | 35.5 |
| T4 | 13 | 17.1 |
| N |  |  |
| N0 | 68 | 89.5 |
| N1 | 8 | 10.5 |
| M |  |  |
| M0 | 73 | 96.1 |
| M1 | 3 | 3.9 |
| **Vital status** |  |  |
| Alive | 37 | 48.7 |
| Dead | 39 | 51.3 |
| **Expression of miR-610** |  |  |
| Low expression | 38 | 50 |
| High expression | 38 | 50 |

**Supplementary Table 2**. Correlation between the clinicopathological features and expression of miR-610

| **Patient characteristics** | | **miR-610 expression** | | ***P*-value** |
| --- | --- | --- | --- | --- |
|  |  | **Low** | **High** |  |
| **Age (years)** | ≤45 | 16 | 15 | 0.356 |
|  | >45 | 22 | 23 |  |
| **Gender** | Male | 35 | 32 | 0.287 |
|  | Female  female | 3 | 6 |  |
| **Clinical stage** | I | 0 | 6 | <0.001 |
|  | II | 2 | 31 |  |
|  | III | 22 | 1 |  |
|  | IV | 14 | 0 |  |
| **T** | T1 | 0 | 5 | <0.001 |
|  | T2 | 2 | 29 |  |
|  | T3 | 23 | 4 |  |
|  | T4 | 13 | 0 |  |
| **N** | N0 | 33 | 35 | 0.455 |
|  | N1 | 5 | 3 |  |
| **M** | M0 | 36 | 37 | 0.556 |
|  | M1 | 2 | 1 |  |
| **Survival time**  **(Median=23 month)** | ≤23 | 15 | 24 | 0.039 |
|  | >23 | 23 | 14 |  |
| **Vital status** | Alive | 21 | 16 | 0.251 |
|  | Dead | 17 | 22 |  |
